# Factors that affect blood eosinophil counts in a non-asthmatic population: Post hoc analysis of data from Brazil

**DOI:** 10.1016/j.waojou.2020.100119

**Published:** 2020-05-16

**Authors:** Namhee Kwon, Emilio Pizzichini, Aruna T. Bansal, Frank C. Albers, Neil Barnes, John H. Rile, Aline Lima-matos, Eduardo Viera ponte, Alvaro A. Cruz

**Affiliations:** a Respiratory Medical Franchise, GlaxoSmithKline, Brentford, Middlesex, UK; b Universidade Federal de Santa Catarina, Florianopolis, Brazil; c Global Medical Expert, Respiratory Franchise, GlaxoSmithKline, UK; d Acclarogen Ltd, St John's Innovation Centre, Cambridge, UK; e Global Respiratory Medical Franchise, GlaxoSmithKline, Research Triangle Park, NC, USA; f William Harvey Institute, Barts and the London School of Medicine and Dentistry, London, UK; g GSK, Medicines Research Centre, Stevenage, Herts, UK; h ProAR, Federal University of Bahia, Bahia, Brazil; i Internal Medicine, Jundiaí Medical School, São Paulo, Brazil

**Keywords:** Atopy, Biomarker, Eosinophil, Normal range, Parasite, AR, allergic rhinitis, BEC, blood eosinophil counts, BMI, body mass index, COPD, chronic obstructive pulmonary disease, EGPA, eosinophilic granulomatosis with polyangiitis, FEV1, forced expiratory volume in 1s, GINA, Global Initiative for Asthma, IgE, immunoglobulin E, IL, interleukin, IQR, interquartile range, ProAR, Program for Control of Asthma in Bahia, SPT, skin prick test

## Abstract

**Background:**

Improved understanding of the normal range of blood eosinophil counts (BEC) and conditions that influence them in non-asthmatic individuals should allow more accurate estimation of the threshold at which eosinophilic disease should be considered, diagnosed, and treated. This analysis investigated the impact of atopy, smoking, and parasitic infection on BEC.

**Methods:**

This was a post hoc analysis of non-asthmatic subjects from a case-control study (CONEP 450/10) conducted at the Program for Control of Asthma in Bahia (ProAR). Participant BECs were measured at baseline; correlations between predefined risk factors and BEC were assessed via univariate and stratified analysis.

**Results:**

Of the 454 participants included, 3% were helminth parasite-positive, 18% were non-helminth parasite-positive; and 450 had BEC data. The median (interquartile range [IQR]) BEC was 152 (96, 252) cells/μL. Any positive skin prick test, elevated total immunoglobulin E, allergic rhinitis, and being a current smoker were all individually associated with higher BEC (p < 0.05) compared with BEC in participants without these factors, but having a non-helminthic parasitic infection was not. Participants with all 4 risk factors that were associated with higher BEC had a median (IQR) BEC of 192 cells/μL (94, 416) versus 106 cells/μL (70, 164) for those with no risk factors.

**Conclusions:**

In non-asthmatic subjects, atopy, allergic rhinitis, and current smoking status were associated with higher BEC compared with subjects without these factors, but BEC values were well below the threshold commonly accepted as normal. Therefore, BEC should be interpreted in the context of an individual's medical conditions and other BEC-influencing factors.

## Background

Blood eosinophil counts have traditionally been used to aid in the diagnosis of atopic diseases and more unusual conditions, such as eosinophilic granulomatosis with polyangiitis (EGPA).^1^^,^^2^ Peripheral blood eosinophil levels above 450 cells/μL are generally considered to be abnormal.^3^ It is now apparent that blood eosinophil count is a marker of response to corticosteroids and anti-interleukin (IL)-5 treatment in a range of airway diseases, particularly asthma and chronic obstructive pulmonary disease (COPD).^4^^,^^5^ Indeed, the Global Strategy for Asthma Management and Prevention (Global Initiative for Asthma [GINA]) guidelines list elevated blood eosinophil count as one of the modifiable risk factors for asthma exacerbations, and they recommend that the presence/absence of elevated eosinophil counts is used to help inform treatment decisions.^6^ Evidence also indicates a relationship between elevated blood eosinophil counts, exacerbations of COPD, and the responsiveness of COPD exacerbations to treatment.^7^, ^8^, ^9^ However, the level of eosinophils considered to be predictive of treatment response in these conditions is considerably lower than the 450 cells/μL threshold cited in the literature.^3^^,^^4^^,^^10^ An understanding of factors that may influence blood eosinophil count is, therefore, of increasing importance.

Currently, data on the normal range of blood eosinophil counts are sparse and are based on population level values, rather than being interpreted in the context of an individual's medical conditions. Importantly, previous studies have shown that factors such as skin reactivity to allergens, allergic disease history, and age can all influence blood eosinophil counts.^11^, ^12^, ^13^, ^14^ In particular, blood eosinophil counts in population-based samples have been shown to correlate with the presence or absence of skin reactivity to common allergens.^11^ In one study, individuals who did not present allergen skin reactivity generally had blood eosinophil counts below 200 cells/μL.^11^ In addition, studies of healthy individuals have reported blood eosinophil counts ranging from 20 to 800 cells/μL.^14^, ^15^, ^16^, ^17^, ^18^ Together, these data suggest that blood eosinophil counts of healthy non-atopic individuals could be substantially different than previously reported.

A greater understanding of the non-serious conditions that may influence blood eosinophil counts should provide a better idea of the threshold at which eosinophilic disease should be considered, diagnosed, and treated. The objective of this analysis was to investigate peripheral blood eosinophil counts in non-asthmatic individuals, and quantify the impact of atopy, rhinitis, smoking, and parasitic infection.

## Methods

### Study design

This was a post hoc analysis of the no asthma control group of a case-control study (CONEP 450/10) of participants from the Program for Control of Asthma in Bahia (ProAR). ProAR is the main public secondary care outpatient reference center specializing in asthma in Salvador City, Brazil. Participants with severe asthma, as well as the following two control groups from the community in which the study outpatient clinic was based, were enrolled between January 2013 and July 2015: (1) participants with mild and moderate asthma; and (2) participants with no asthma. The study was approved by the Institutional Review Board of the Federal University of Bahia and the Brazilian National Review Board, validating its compliance with the Declaration of Helsinki 2013 on ethical principles for medical research involving human subjects. All participants provided informed consent.

### Participants

This post hoc analysis included participants from the non-asthma group of the ProAR study, i.e., those who were ≥18 years of age with no history of asthma or other lung disease, were not pregnant, and were underprivileged users of the public health system of Salvador, Northeastern Brazil, a large urban center. They were recruited by poster advertisement in public places and on transportation, followed by telephone screening and invitation.

### Endpoints and assessments

The co-primary endpoint of the original ProAR study was blood eosinophil counts in participants with severe asthma, to be compared with those of participants with mild-moderate asthma and participants without asthma. In this post hoc analysis, participant demographics and baseline characteristics, including blood eosinophil counts determined during the original study, were analyzed in non-asthmatic participants. Peripheral blood samples were collected at the baseline study visit, and peripheral blood cells including eosinophils and neutrophils were measured using automated equipment (Cell-Dyn Ruby, Abbott, Maidenhead, Berkshire, UK) in a period between 2013 and 2015. In order to minimize variation and standardize the collection of the peripheral blood cell count data, all measurements were performed in the same laboratory within the Federal University. At the same visit, all participants were assessed for the following predefined risk factors for high blood eosinophil counts: positive skin prick test (SPT; yes/no); elevated serum immunoglobulin E (IgE; <70 IU/mL/≥70 IU/mL); allergic rhinitis (yes/no); any helminth infection or non-helminth parasitic infection (assessed by 1 stool examination; yes/no); current smoking (smoked in the last 30 days; yes/no); reversible airflow limitation (increase in forced expiratory volume in 1s [FEV_1_] ≥12% and ≥200 mL; yes/no); and high body mass index (BMI; 25–29.9 kg/m^2^ [yes/no] or ≥30.0 kg/m^2^ [yes/no]). Univariate and stratified analyses were performed to determine the influence of these risk factors on blood eosinophil count. All participants positive for helminth infection or with a missing data point for 1 or more stratifying factors were excluded from the stratified analysis.

### Statistical analysis

For the univariate analysis, putative risk factors were assessed singly for association with blood eosinophil count using a Kruskal–Wallis test, and data were displayed using box plots. Odds ratios, both unadjusted and adjusted for age, were also calculated for associations between individual risk factors and blood eosinophil count. For the stratified analysis, pairwise correlation between risk factors was assessed by means of Pearson's correlation coefficient. Combinations of 2, 3, and 4 risk factors associated with eosinophil count were considered jointly. Median blood eosinophil counts were calculated for different combinations of risk factors. Distributions were displayed in a jitter plot. For ease of reading, blood eosinophil counts were log-transformed in some graphical displays.

## Results

### Participant population

A total of 454 participants from the non-asthmatic control group of the original ProAR study were included in this post hoc analysis. Of the 418 participants who had a stool sample available, 13 (3%) were positive for helminth infection and 77 (18%) were positive for a non-helminth parasitic infection (Table 1). A full list of parasites identified is included in Table S1. A greater proportion of participants with parasitic compared with non-parasitic infections were female, had a positive SPT result and had higher IgE levels.

**Table 1:** Participant demographics and characteristics in non-asthmatic participants with a stool sample available (N = 418)

	Total (N = 418)	No parasite (N = 328)	Parasite positive, excluding helminth (N = 77)	Helminth positive (N = 13)
**Age, years, mean (SE)**	45 (0.6)	45 (0.7)	46 (1.5)	40 (2.4)
**Female, n (%)**	358 (85.7)	287 (87.5)	62 (80.5)	9 (69.2)
**BMI, kg/m**^**2**^**, mean (SE)**	27.3 (0.3)	27.4 (0.3)	26.8 (0.6)	26.0 (1.1)
**BMI,** ≥ **30 kg/m**^**2**^**, n (%)**	111 (26.6)	91 (27.7)	17 (22.1)	3 (23.1)
**Smoking history**				
Ever smoked, n (%)	115 (27.6)^a^	93 (28.4)^d^	17 (22.1)	5 (38.5)
Current smoker, n (%)	34 (8.2)^a^	27 (8.3)^d^	5 (6.5)	2 (15.4)
Former smoker, n (%)	81 (19.4)	66 (20.2)^d^	12 (15.6)	3 (23.1)
Pack/year, median (IQR)	6 (1, 17)^b^	6 (1, 17)^e^	5 (0, 20)^h^	7 (6, 12)^l^
**Allergic rhinitis, n (%)**	79 (23.0)^c^	63 (23.5)^f^	12 (18.5)^i^	4 (36.4)^m^
**Gastro-esophageal reflux disease, n (%)**	26 (6.2)	20 (6.1)	4 (5.2)	2 (15.4)
**SPT positive to any aeroallergen, n (%)**	113 (32.9)^c^	86 (32.1)^f^	22 (33.9)^i^	5 (45.5)^m^
**IgE total**				
Geometric mean (IQR)	96.5 (32, 337)	88.1 (28, 334)^g^	126.4 (52, 322)^j^	196.1 (69, 487)
Median (IQR)	118.6 (32, 337)	116 (28, 334)^g^	164 (52, 322)^j^	115 (69, 487)
**Eosinophils**				
Geometric mean (IQR)	147.0 (94, 248)	150.2 (94, 250)^d^	126.9 (89, 231)^k^	205.2 (162, 274)
Median (IQR)	151 (94, 248)	149 (94, 250)^d^	149 (89, 231)^k^	180 (162, 274)

BMI, body mass index; IgE, immunoglobulin E; IQR, interquartile range; SE, standard error; SPT, skin prick test.

a N = 417;

b N = 114;

c N = 344;

d N = 327;

e N = 92;

f N = 268;

g N = 319;

h N = 17;

i N = 65;

j N = 76;

k N = 75;

l N = 5;

m N = 11

### Blood eosinophil counts

**Fig. 1:**
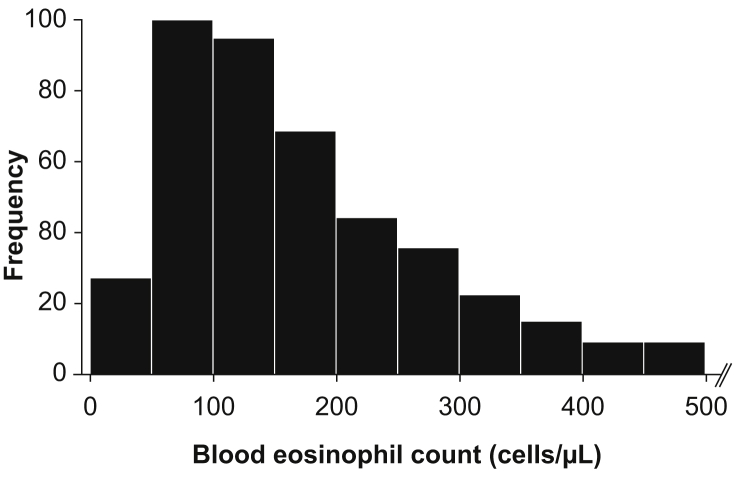
Distribution of blood eosinophil counts in non-asthmatic participants (N = 450)

A total of 450 non-asthmatic participants had blood eosinophil data available. The distribution of blood eosinophil counts in this group is shown in Fig. 1. Due to the skewed distribution of the data, the interquartile range (IQR) was used to describe the results and provide a notion of where the normal range may be, rather than using the 95% confidence interval, as for normally distributed data. The median (IQR) blood eosinophil count in the overall group was 152 (96, 252) cells/μL, with 5th and 95th percentiles of 50 and 508 cells/μL, respectively.

### Risk factors for increased blood eosinophil count

**Fig. 2:**
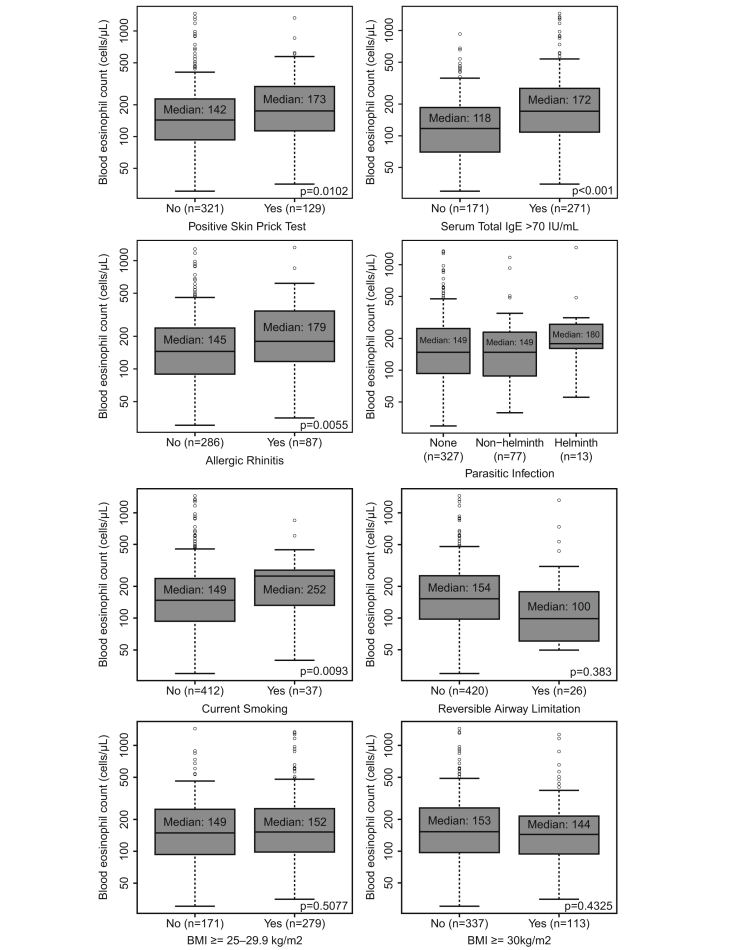
Univariate analysis of the effect of individual risk factors on log-transformed blood eosinophil counts in non-asthmatic participants. BMI, body mass index; IgE, immunoglobulin

A positive SPT, elevated total IgE, comorbid allergic rhinitis, and being a current smoker were all associated with higher blood eosinophil counts (p < 0.05) in the univariate analysis (Fig. 2). In particular, median (IQR) blood eosinophil counts in participants with a positive SPT versus negative SPT were 173 (112, 295) cells/μL vs 142 (92, 225) cells/μL (p = 0.0102). In participants with an IgE ≥70 IU/mL, median (IQR) blood eosinophil count was 172 (109, 284) cells/μL compared with 118 (70, 186) cells/μL (p < 0.001) in those with IgE <70 IU/mL. Blood eosinophil counts were 179 (116, 342) cells/μL and 145 (89, 238) cells/μL in participants with and without allergic rhinitis, respectively (p = 0.0055). Median (IQR) blood eosinophil counts in current smokers versus those not currently smoking were 252 (133, 287) cells/μL versus 149 (94, 238) cells/μL (p = 0.0093). A non-significantly higher blood eosinophil count was observed in participants with no airway reversibility than in those with airway reversibility (154 [99, 254] cells/μL vs 100 [63, 172] cells/μL, respectively [p = 0.383]). BMI and parasitic infection did not appear to be associated with blood eosinophil counts. The low number of participants with helminth infections (n = 13) did not afford sufficient power to allow accurate estimates and comparisons of blood eosinophil counts in this subpopulation. However, it is noteworthy that the median (IQR) blood eosinophil count among these 13 participants was 180 (162, 274) cells/μL. Associations between individual risk factors and blood eosinophil counts were unchanged following adjustment for participant age.

**Fig. 3:**
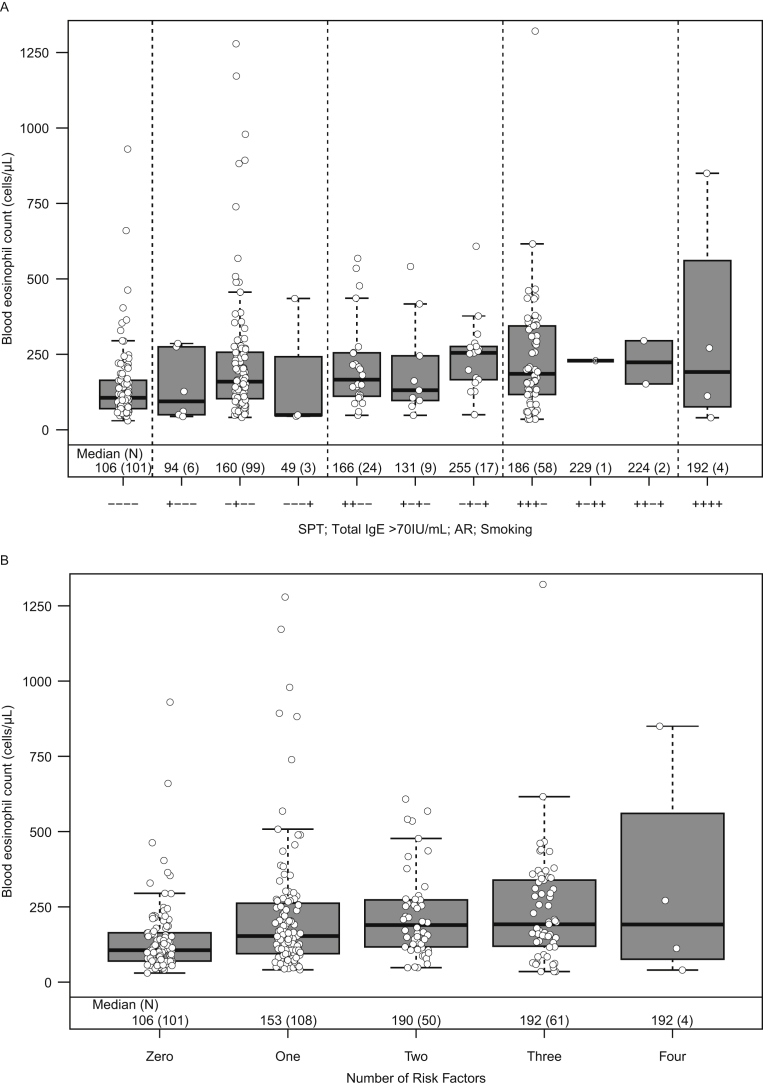
Stratified analysis of the effect of risk factor combinations on blood eosinophil count in non-asthmatic participants stratified by risk factor (A), and by cumulative number of risk factors (B). N = 324; all participants positive for helminth infection (n = 13) or with a missing data point for one or more stratifying factors (n = 81) were excluded from the stratified analysis; black bars represent median values and boxes represent IQR. AR, allergic rhinitis; IgE, immunoglobulin E; IQR, interquartile range; SPT, skin prick test

Blood eosinophil count distributions for different combinations of risk factors that were associated (p < 0.05) with higher blood eosinophil counts in the univariate analysis were compared in the stratified analysis (Fig. 3). In total, 324 participants had complete data for all 4 factors (SPT, allergic rhinitis, high total IgE and current smoking) identified from the univariate analysis and were included in the stratified analysis. Participants who were positive for all 4 risk factors had a median (IQR) blood eosinophil count of 192 cells/μL (94, 416) compared with 106 cells/μL (70, 164) in participants with none of these risk factors (Fig. 3). The median blood eosinophil count ranged from 49 to 160 cells/μL in participants with one risk factor, 131 to 255 cells/μL in those with 2 risk factors, and 186 to 224 cells/μL in those with 3 risk factors. In addition, positive but weak correlations were seen between high total IgE and SPT positivity (*r* = 0.3305), and positive strong correlations were found between allergic rhinitis and SPT positivity (*r* = 0.7791) (Table S2).

## Discussion

This analysis aimed to determine the impact of atopy and other conditions, such as smoking, overweight/obesity and parasitic infection on blood eosinophil counts in non-asthmatic individuals. Such mild medical conditions are not usually considered when a blood eosinophil count is reviewed, either in sick patients or in otherwise healthy people. In line with previous reports, our results indicated that a positive SPT was associated with elevated blood eosinophil counts.^11^^,^^12^ Additionally, we found that elevated IgE (≥70 IU/mL), allergic rhinitis and smoking were similarly associated with elevated blood eosinophil counts. In participants who were negative for all 4 of these risk factors, the median (IQR) blood eosinophil count was 106 cells/μL (70, 164), while in those positive for all 4 risk factors the median blood eosinophil count was 192 cells/μL (94, 416).

Elevated eosinophil counts are associated with a number of pathological disorders, including severe asthma, EGPA, and hypereosinophilic syndrome.^19^, ^20^, ^21^ To allow for the accurate identification of eosinophil count abnormalities, it is important to understand the normal range of blood eosinophil counts, both in the general population and in patients with asthma. Here we focus on eosinophil levels in the general population. Generally, the 95th percentile value is often used as the upper range of normal in data with a normal distribution. For skewed data, it is deemed more appropriate to use the IQR to represent the data better and avoid outliers influencing data interpretation. Indeed, it is likely that this approach would be appropriate for a number of biomarkers for which skewed data have been demonstrated. As the distribution of blood eosinophil counts was skewed in the current study, the IQR was used to provide a rough idea of where the normal range is likely to be. Based on this method, as the 75th percentile in this study population was found to be 252 cells/μL, the upper limit of the normal range of blood eosinophils may be much lower than the accepted 450 cells/μL upper limit, beyond which individuals are generally considered to have elevated blood eosinophils.^3^ Additionally, the IQR identified in this population (96–252 cells/μL) is much lower than the 270–800 cells/μL upper limit reported in previous population-based studies, assessing normal blood eosinophil counts in a range of different ethnic populations.^14^, ^15^, ^16^, ^17^, ^18^^,^^22^

Eosinophils perform several functions in the innate immune response, including tumor immune surveillance and adipose tissue regulation, and they have been implicated in primary effector mechanisms against parasitic invasion.^23^, ^24^, ^25^ Therefore, we also sought to determine the effect of parasitic infections on blood eosinophil counts. The presence of non-helminth parasitic infections was not associated with elevated blood eosinophil count; however, consistent with a previous report, we observed a trend for an association between helminth infection and elevated blood eosinophil count (180 cells/μL compared with 152 cells/μL in the overall study population).^26^ Unfortunately, further univariate and stratified analyses to determine the association between blood eosinophil count and these parasites were not possible due to the small number of participants with helminth infection (n = 13; 3%). This figure is substantially lower than the 13–>20% of the Brazilian population estimated to be at risk of helminth infection based on previous reports.^27^^,^^28^ It is possible that the use of only 1 stool sample may not have been sufficient to detect helminth infection, potentially contributing to the low levels in our analysis. However, it has been suggested that a comprehensive examination of a single stool sample is sufficient to detect parasite infection in the majority of patients,^29^ indicating that the use of stool for parasitic identification is unlikely to have significantly influenced the frequency of helminth infection. Furthermore, as blood eosinophil counts were far lower than the currently accepted normal level, even among subjects infected with non-helminth parasites, it is likely that few helminth infections were missed. Indeed, the low rate of helminth infection found here, compared with rates shown in previous studies, may be attributed to improvement in sanitation in the urban environment in which this study was conducted.

Previous studies have identified additional factors that may influence blood eosinophil counts, including age, gender, and ethnicity.^15^, ^16^, ^17^, ^18^ In particular, a significant 50 cell/μL mean difference in blood eosinophil counts between African descent (100 cells/μL) and Indian (150 cells/μL) participants (p < 0.05), but not between African descent and caucasian participants, has been reported.^15^ In addition, a separate study reported significant differences in eosinophil counts between males and females of different ethnic origin (Afro-Caribbean, African, and Caucasian).^16^ As the population of Salvador in Brazil has a strong African descent-ethnic mix, these previous studies may suggest a need for caution in generalizing our findings to other populations. However, the mean eosinophil count in the European Unbiased BIOmarkers in PREDiction of respiratory disease outcomes (U-BIOPRED; NCT01866306) population (143 cells/μL; unpublished data) was similar to the mean value calculated in the present study. Furthermore, as previous studies did not determine the underlying factors for their results, it is not possible to determine if the reported ethnic differences were due to confounding environmental factors or genetic differences.

There are several strengths of this analysis. The sample size of 454 participants selected from the community is fairly large and allowed for both univariate and stratified testing of potential factors influencing blood eosinophil count. Moreover, blood eosinophil counts were determined using a validated tool (Cell-Dyn Ruby), ensuring their accuracy. In addition, 418 participants had a stool sample available to determine the presence or absence of parasitic infection, most subjects provided information on smoking and a history of chronic rhinitis, underwent SPT, and had total IgE measurements, allowing for these potential risk factors to be examined for their influence on blood eosinophil counts. Limitations included the post hoc nature of the analysis, meaning that comparisons could have lacked adequate statistical power. In the original study, a fully random sample of the general population was not obtained, although the recruitment strategy meant that it was likely to be representative as participants were recruited from the community, invited by public advertisement and screened at a specific call center. In addition, it should be noted that there may be many different causes for an increased blood eosinophil count, and indeed it is a hallmark of several diseases.^30^ Furthermore, blood eosinophils are only one of several biomarkers of a type 2 response.^31^ Nonetheless, blood eosinophil counts are readily available and have been used in multiple studies as an indicator of a type 2 immune response.^32^^,^^33^ Participant blood eosinophil counts were also only determined at a single time point i.e., the baseline visit. Previous studies have demonstrated that blood eosinophil counts are broadly stable at the population level over time in both asthma and COPD,^34^^,^^35^ although a small degree of within-individual variability has been reported due to a range of factors including eating, exercise, medication, and time of testing.^36^ Overall, this suggests that a single measurement of blood eosinophil count is sufficient for determining the normal eosinophil range across a larger population.

## Conclusions

In conclusion, this analysis demonstrates that atopy, as indicated by a positive SPT to common aeroallergens, higher total IgE or allergic rhinitis, and current smoking are associated with elevated blood eosinophil counts. Our study, however, was not intended to propose that blood eosinophil counts should be used as a diagnostic tool; instead, it aimed to help us better understand the cutoffs for blood eosinophil counts as biomarkers of a type 2 inflammatory response in asthmatic patients using evidence from non-asthmatic subjects. For non-asthmatic subjects, in this study, blood eosinophil counts were still considerably lower than the current perceptions of the "normal range", suggesting that the normal range of blood eosinophil counts may be overestimated. However, these findings should be interpreted with caution as they may not be generalizable to the wider asthma population. Nonetheless, even when more sophisticated diagnostic tools are available, measuring blood eosinophil counts is an easy technique for physicians. However, when interpreting the thresholds for blood eosinophil counts physicians should consider these in a disease-specific context, taking into account an individual's medical history and association with any known factors that influence blood eosinophil counts.

## Funding

This post hoc analysis and the parent study (200066) were funded by GSK and the 10.13039/100013101National Research Council for Brazil (10.13039/501100003593CNPq).

## Consent for publication

N/A: identifiable patient data are not included.

## Competing interests

NK, EP, FCA, NB and JHR are employees of GSK and hold stocks/shares. AAC is an employee (faculty member) of ProAR – Federal University of Bahia, Brazil, received investigator-initiated funding from GSK (200,066) and the 10.13039/100013101National Research Council of Brazil (10.13039/501100003593CNPq), and has received personal fees from Astrazeneca, 10.13039/100001003Boehringer Ingelheim, 10.13039/100007560Chiesi, Eurofarma, GSK, MEDA, 10.13039/100004336Novartis, and 10.13039/100004339Sanofi. ATB is a paid consultant of GSK. EVP and ALM are associate investigators of ProAR, Federal University of Bahia, Brazil.

## Ethics approval

The study was approved by the Institutional Review Board of the Federal University of Bahia and the Brazilian National Review Board, validating its compliance with the Declaration of Helsinki 2013 on ethical principles for medical research involving human subjects. All participants provided informed consent.

## Authors’ contributions

NK and NB made substantial contributions to the conception and design of this post-hoc analysis; AAC, AL-M and EP were involved in the acquisition of data in the original study; all authors were involved in data analysis and interpretation, drafting the work, and revising it critically for important intellectual content. All approved the final version to be published and agree to be accountable for all aspects of the work.

## Submission statement

None of the data of this analysis has been published or is under consideration for publication elsewhere.
