# Supplementary material for: Factors that affect blood eosinophil counts in a non-asthmatic population: Post hoc analysis of data from Brazil
Source: World Allergy Organ J. 2020 May 16;13(5):100119. doi: 10.1016/j.waojou.2020.100119 (PMC7232113; doi:10.1016/j.waojou.2020.100119)
Supplement: Multimedia component 1 [file mmc1.docx]

**Supporting Information**

**Factors that affect blood eosinophil counts in a non-asthmatic population: post hoc analysis of data from Brazil**

**Contents**

Table S1. Parasitic infections identified in non-asthmatic participants.

Table S2. Pearson correlations between putative risk factors in non-asthmatic participants.

**Table S1.** Parasitic infections identified in non-asthmatic participants.

| **Infections** | **Non-asthmatic participants (N=454)** |
| --- | --- |
| **Helminth, n (%)** |  |
| *Schistosoma mansoni* | 5 (1) |
| *Ascaris lumbricoides* | 4 (<1) |
| *Trichuris trichiura* | 3 (<1) |
| *Ancylostoma* | 1 (<1) |
| **Other parasitic infections, n (%)** |  |
| *Endolimax nana* | 65 (14) |
| *Entamoeba coli* | 37 (8) |
| *Iodamoeba bütschlii* | 4 (<1) |

**Table S2.** Pearson correlations between putative risk factors in non-asthmatic participants.

|  | **SPT** | **Total IgE** | **Allergic**  **rhinitis** | **Parasite** | **Smoking** | **Reversible airflow obstruction** | **BMI  25–29.9 kg/m^2^** | **BMI  ≥30 kg/m^2^** |
| --- | --- | --- | --- | --- | --- | --- | --- | --- |
| **Total IgE** | 0.3305 | - | - | - | - | - | - | - |
| **Allergic rhinitis** | 0.7791 | 0.2550 | - | - | - | - | - | - |
| **Helminth** | 0.0494 | -0.0230 | 0.0591 | - | - | - | - | - |
| **Smoking** | -0.0580 | 0.0859 | -0.0421 | 0.0618 | - | - | - | - |
| **Reversible airflow obstruction** | 0.0921 | 0.0169 | 0.1329 | -0.0470 | -0.0337 | - | - | - |
| **BMI 25–29.9 kg/m^2^** | 0.0112 | -0.0388 | 0.0533 | -0.0434 | -0.0047 | 0.0069 | - | - |
| **BMI ≥30 kg/m^2^** | -0.0959 | 0.0884 | -0.1076 | 0.0003 | 0.0028 | -0.0125 | -0.4853 | - |
| **Any parasite** | 0.0406 | 0.0402 | -0.0171 | 0.3455 | -0.0124 | -0.1361 | 0.0057 | -0.0509 |

BMI, body mass index; IgE, immunoglobulin E; SPT, positive skin prick test.
